# Assessing and teaching job-related social skills to adults with neurodevelopmental disorders in Italy

**DOI:** 10.1007/s40617-023-00873-2

**Published:** 2023-11-06

**Authors:** Claudio Radogna, Guido D’Angelo, Dorothea C. Lerman

**Affiliations:** 1 Cooperativa Dalla Luna, Bari, Italy; 2 Disability and Health Integrated Program, Local Health Unit, Bologna, Italy; 3 University of Houston-Clear Lake, BCBA-D, 2700 Bay Area Blvd., MC 245, Houston, TX 77058 USA

**Keywords:** Employment, Job-related social skills, Neurodevelopmental disabilities

## Abstract

Social challenges in the work place can serve as an obstacle to regular employment for many individuals with neurodevelopmental disabilities (NDD). Nonetheless, few studies have focused on interventions to improve job-related social skills or included residents of countries outside of the United States. This study replicated and extended prior research by evaluating the acquisition of job-related social skills with three individuals with NDD residing in Italy. Results suggested that a package consisting of behavioral skills training and token reinforcement was effective for teaching the skills in the clinic and in extension to real work contexts. Furthermore, social validity surveys indicated that the participants, professionals, and caregivers of individuals with NDD considered the skills and interventions to be acceptable. These findings have implications for improving employment outcomes for individuals with NDD across the globe.

Regular employment can play an essential role in an individual’s quality of life, permitting independence, creating social opportunities, and contributing directly to physical and psychological well-being (Bennett & Dukes, 2013; Brand, 2015; Fleming et al., 2013; García-Villamisar et al., 2002; McKee-Ryan et al., 2005; Taylor et al., 2014). However, a large proportion of people with neurodevelopmental disabilities (NDD) who want to work have difficulty obtaining and maintaining employment (Hendricks, 2010). Such difficulties have been reported globally, with research in the United States, Europe, Australia, and Asia suggesting that less than 50% of individuals with autism spectrum disorder (ASD) are employed (e.g., Cameron et al., 2022; Howlin & Mash, 2012; Maslahati et al., 2022; Roux et al., 2013; Wei et al., 2015; Zhou et al., 2022).

Employment outcomes appear to be particularly poor for individuals with ASD. In the United Kingdom, for example, individuals with ASD reportedly have the lowest employment rate among individuals with disabilities between the ages of 16 and 65 years (22% vs 54%; Office for National Statistics, 2022). In a presentation to the Committee on Employment and Social Affairs of the European Parliament, Autism-Europe estimated that fewer than 10% of individuals with ASD have been employed, a rate dramatically below that of people with and without other disabilities (Baranger, 2019). Regardless of country, both individuals with ASD and their communities benefit when improved employment outcomes increase the independence and well-being of this population.

Research suggests that difficulties in social and communication skills may be a key barrier in achieving successful employment (e.g., Greenspan & Shoultz, 1981; Hendricks, 2010; Mueller, 1988; Wei et al., 2015). Among other social demands, employees must ask for help when needed, respond appropriately when asked to improve their work, and notify the supervisor when they encounter problems (Montague et al., 2017; Partington & Mueller, 2015). Research has identified a number of effective behavioral interventions for improving social-communication skills (e.g., Radley et al., 2020). However, few studies have focused on the social skills specific to success on the job (e.g., using small talk with colleagues, asking for help, apologizing for unintentional mistakes) or included direct measures of these behaviors in their outcomes (Gorenstein et al., 2020). In the majority of research on job-related skills training, experimenters targeted general occupational skills, such as remaining on task (e.g. Montgomery et al., 2011; Watanabe & Sturmey, 2003), completing specific jobs (e.g., Kellems & Morningstar, 2012), and interviewing for jobs (Morgan et al., 2014). In addition, the majority of these studies have been conducted with residents of the United States despite the global relevance of employing people with neurodevelopmental disabilities (Barbaro & Shankardass, 2022).

Lerman et al. (2017) developed a methodology for assessing job-related social skills in a clinic setting, noting that some professionals may not have access to job sites when preparing individuals with NDD for employment. The experimenters arranged opportunities for the participants to engage in a variety of social skills, such as asking for help with missing or nonfunctioning materials, confirming understanding of instructions, and responding appropriately to corrective feedback, within the context of authentic work situations at the clinic. Results indicated that the assessment provided an efficient way to identify appropriate targets for instruction.

In a subsequent study, Grob et al. (2019) completed an initial assessment of job-related social skills similar to that described by Lerman et al. (2017) with three individuals diagnosed with NDD. After identifying at least three targets for each participant, the experimenters evaluated the effectiveness of brief behavioral skills training (BST) combined with stimulus prompts to promote generalization of the skills to nontraining settings. If the participant did not demonstrate an improvement with the initial intervention, the experimenter introduced a series of increasingly more intensive interventions, including feedback and monetary reinforcement, until the participant met a mastery criterion. Results showed that BST plus stimulus prompts was effective for two of the three participants. The third participant did not meet the mastery criterion for most of the skills until the experimenter introduced monetary reinforcement, which was subsequently faded successfully. The skills generalized to a nontraining context for all participants. Although these results are promising, the experimenters did not evaluate the skills within the context of actual work settings. Intervention in real work environments involving the employer and tasks similar to those that the trainee might encounter on the job can be particularly fruitful in building a repertoire of skills that are immediately applicable to professional contexts (Anderson et al., 2017).

Further research is needed on effective strategies for preparing individuals with ASD to respond to social demands in the workplace. It is particularly important to replicate this work with individuals residing outside of the United States to establish the cultural relevance of these targets and commonly used behavior-analytic procedures. To date, no studies on teaching job-related social skills have included individuals with NDD in Italy.

Thus, the purpose of this study was to replicate and extend Lerman et al. (2017) and Grob et al. (2019) by assessing and teaching job-related social skills to three individuals with NDD residing in Italy. After conducting an assessment similar to that described in the prior studies to identify targets for intervention, the experimenter evaluated the efficacy of a training package consisting of BST and token reinforcement. Finally, the generality of the training was evaluated via extension to real work contexts, with instructions and tasks presented by an employer rather than by an experimenter.

## Method

### Participants

Participants were three young adults, aged 19 to 25 years. Each had earned a high school diploma and were unemployed at the time of the study; all participants spoke Italian as their primary language. They were recruited from a center for autism and other developmental disabilities located in southern Italy, where they received training to improve adaptive behavior and social skills. The families of five individuals who showed deficits in job-related social skills and who ranged in age from 19 to 28 years received an invitation letter, addressing the goals, requirements, and study procedures. Three of the families indicated an interest in participating. At the time of the study, none of the participants had been previously employed or had received training on job-related social skills, although all had received some job-specific training or experience.

Alberto was a 19-year-old man diagnosed with autism spectrum disorder (Asperger's Disorder, *DSM IV-TR*; American Psychiatric Association, 2000). He had received a full-scale IQ score of 84 on the Wechsler Adult Intelligence Scare-Revised (WAIS-R; Wechsler, 1981) and lived at home with his parents at the time of the study. He spoke in complete sentences. During his last 3 years of high school, he participated in a program that included fieldwork experience at primary schools (kindergarten) in his local community. For his fieldwork experience, he observed teachers and recorded notes about his observations. He did not receive any training.

Maria was a 22-year-old woman diagnosed with attention deficit and hyperactivity disorder (combined subtype), oppositional-defiant disorder, and intellectual disability. She had received an IQ score of 36 on the Leiter International Performance Scale (Leiter-R; Roid & Miller, 1997) and lived at home with her parents and her sister at the time of the study. Maria spoke in complete sentences. Prior to the study, Maria had assisted a secretary during a professional workshop. Her responsibilities included greeting the workshop participants and filing and folding documents, but she did not require any training to complete these activities.

Giacomo was a 25-year-old man diagnosed with Fragile X Syndrome and intellectual disability. He had received an IQ score of 45 on the Leiter-R and lived at home with his parents and sister at the time of the study. Giacomo’s spoke in complete sentences, but his articulation was poor, and he stuttered occasionally. Prior to the study, he received job training (unpaid) as a warehouse worker and stocker at a supermarket and as a farmer (e.g., sorting and packing fruits and vegetables) at an agriculture project company that was managed by a local social cooperative.

### Setting and Materials

All baseline and initial training sessions were conducted in rooms usually dedicated to behavior therapy, consultation, and assessment at the center. The room was approximately 5 m × 4 m and was equipped with a desktop computer, a printer, a computer desk, chairs, a shredder, and a filing cabinet. Each room had a camera mounted on the ceiling. The majority of sessions were recorded for data collection purposes. Some of Alberto’s sessions also were conducted in his bedroom or the living room of his home. Alberto’s bedroom was approximately 3 m × 3 m and contained a table, a chair, a wardrobe, a bed, a bookshelf and two wall shelves. His living room was approximately 10 m × 4 m and contained a large table, six chairs, two sofas, three bookshelves, and equipment and materials needed to complete the assigned work activities (e.g., copier, printer, binders, stapler, clothes). Sessions typically occurred 2 days per week. The same experimenter served as the supervisor throughout the study. During some sessions, the supervisor was in the room with the participant, but he stood at least 3 m away from the participant and engaged in other activities. In other sessions, he was located outside of the session room but in random areas of the building.

Following the initial training, additional sessions were conducted in real work settings that included a psychotherapy office, a toy and book store for children, and a professional school for catering. The purpose was to evaluate the generality of the teaching procedures by extending the training to contexts beyond a controlled clinic setting. Assigned work tasks were different from those in the training setting. At the psychotherapy office, the assigned tasks included cleaning, arranging books and manuals, and assembling furniture. In the toy and book store, tasks included cleaning, stocking books and toys, arranging materials on shelves, and sticking price tags on materials. At the professional school for catering, tasks included serving tables, cleaning, using a coffee machine, and arranging food and items on a bar counter. The owner or supervisor in that setting served as the supervisor during the sessions, with the exception of sessions that occurred in the psychotherapy office (see further description below), where the supervisor was an experimenter who was not involved in the baseline and training sessions. The experimenter also was present in the room but stood at least 4 m from the participant.

#### Response Measurement, Reliability, and Procedural Integrity

Previously trained observers collected data on the occurrence or nonoccurrence of participant behaviors as described by Grob et al. (2019). The behavior was scored as occurred if the participant emitted the target response each time an evocative situation was presented. Target responses included (1) confirm understanding; (2) ask for help; (3) notify of task completion; (4) ask for task assignment; and (5) respond to corrective task feedback. A comprehensive list of targeted responses and definitions are displayed in Table 1, along with the associated evocative situations (described further below). Semantic, syntactic, and grammatical variations of targeted vocal responses were scored as correct. For the assessment, data on participant behavior were expressed as a percentage of opportunities by dividing the number of correct responses by the total number of opportunities provided during the assessment and converting to a percentage. For the remaining phases of the study, data on participant behavior were expressed as a percentage of opportunities per session. About 80% of the sessions were videorecorded for the purpose of calculating interobserver agreement and procedural integrity data when it was not possible to have a second observer present during the sessions.

**Table 1:** Targeted Behaviors, Associated Evocative Situations, and Definitions

Behavior (Evocative Situation)	Definition
Confirm Understanding (Clear Instructions)	Emitting statements or manding for information while repeating part of the instruction given by the supervisor to confirm understanding of the instruction or assigned task (e.g., "Okay, I am going to fold my shirts," "So, you want me to sort the cards by color, right?") within 5 s of an instruction; excluded simply nodding or saying “okay.”
Ask for Help (Vague Instructions)	Emitting statements or manding for information that would lead the supervisor to explain or model how to complete the entire task (e.g., "Can you tell me/show me how?" "I did not get it.") within 5 s of the instruction.
Ask for Help (Task Not In Repertoire)	Emitting statements that refer to the participant’s inability to complete the task (e.g., "I don't know how to create a bar graph") or manding for information that would lead the supervisor to explain or model how to complete the task correctly (e.g., "Can you show me what to do?"); must be emitted after no more than 1 min of off-task behavior or 5 min of working unsuccessfully on the assigned task.
Ask for Help (Missing or Broken Materials)	Manding for materials (e.g., "I need more napkins") or assistance with nonfunctioning materials (e.g., "This cutter does not work"); must be emitted no more than (a) 1 min of off-task behavior; (b) 5 min after working unsuccessfully on the assigned task; or (c) 5 min after unsuccessfully searching for the needed materials.
Notify of Task Completion (Task Completed)	Emitting statements that indicate the task was completed (e.g., “I’m done”) no more than 1 min after completing the task.
Ask for Task Assignment (Task Completed)	Manding for information about the next task assignment immediately after notifying supervisor of task completion (in the absence of task feedback) (e.g., “What next?”); excluded independently engaging in another task.
Respond to Corrective Task Feedback (Clear or Vague Corrective Feedback)	Apologizing, defined as emitting a statement of remorse (e.g., "I’m sorry," "I will pay more attention."); asking for clear feedback if appropriate (e.g., “What did I do wrong?”); confirming understanding (e.g., "I understand, I'll fix it.”); and correcting the assigned task as described by the supervisor.

A second observer independently collected data on the dependent variables for at least 33% of the sessions for each participant, across evocative situations. Interobserver agreement (IOA) was calculated for each dependent variable by dividing the number of agreements by the number of agreements and disagreements, for each opportunity to emit the target response, and converting the result into a percentage. Mean agreement for all responses across assessment, training and generalization was 97% for Alberto (range: 95%–100%), 99% for Maria (range: 97%–100%), and 96% for Giacomo (range: 92%–100%). The independent observer also assessed, through the use of checklists shown in Table 2, whether the experimenter implemented the following behaviors correctly or incorrectly during at least 33% of the sessions for each participant: (1) delivered the task instruction (clear or vague); (2) responded to participant's requests for help or task completion statements; (3) delivered feedback (clear or vague); (4) provided broken, missing, or insufficient materials; and (5) concluded the work task. The number of components scored as correct was divided by the number of correct and incorrect components for each evocative situation and converted to a percentage. Mean procedural integrity was 99% for Alberto (range: 93%–100%), 96% for Maria (range: 86%–100%), and 97% for Giacomo (range: 86%–100%).

**Table 2:** Procedural Integrity Checklist

Phase	Integrity check
*Assessment*	1. Before starting, the experimenter provides the participant with a general description of the assessment.
	2. The experimenter presents a specific evocative situation (as defined in the procedure) to evoke the target response.
	3. The experimenter waits 5 s before leaving the room or walking away.
	4. The experimenter provides feedback or ends the activity according to the criteria written in the procedure.
	5. The experimenter collects data on the appropriate data sheet.
	6. The experimenter provides neither reinforcement nor verbal praise based on the participant's performance.
	7. The experimenter alternates the different evocative situations to prevent the same situation in two consecutive trials.
*Behavioral Skill Training*	1. During training, the experimenter engages in behavioral skill training (BST).
	*2. Instruction*: The experimenter provides a rationale of the skill and explains of to perform the skill.
	3. *Model*: The experimenter models the skills.
	4. *Practice*: The experimenter asks the participant to perform the skill.
	5. *Feedback*: The experimenter provides feedback on the performance.
	6. If the performance is correct, the experimenter praises the participant.
	7. If the participant did not perform the task correctly, the experimenter represents the "practice" phase (for a maximum of three times; otherwise they suspend the teaching and revise the procedure).
*Training*	1. The experimenter informs the participant that every time they correctly perform the target behavior, they will get a token point.
	2. The experimenter presents a specific evocative situation.
	3. In case of a correct response, the experimenter immediately reinforces the performance with descriptive feedback, verbal praise, and tokens.
	4. If the participant does not emit the target behavior, the experimenter represents BST from the "modeling" phase.
	5. The experimenter reinforces the performance with only verbal praise if modeling occurred before the target response.
	6. The experimenter collects data.
*Extension*	1. The sessions are conducted in a real-world work or training environment.
	2. The experimenter presents a BST booster session only before the first session.
	3. The evocative situations are presented by job supervisor.
	4. The experimenter does not interact with the participant.

### Procedures

#### Assessment (Baseline)

The purpose of this phase was to identify intervention goals for each participant and to obtain baseline data on the targeted skills by replicating the assessment procedures described by Lerman et al. (2017) and Grob et al. (2019). To prepare for the assessment, the experimenter met with the participants and their caregivers to identify work tasks that likely were and were not in the participants’ repertoire. The experimenters also obtained copies of the participants’ individual educational plans and other psychological reports and assessments.

Each assessment was conducted across a minimum of 3 separate days, during an approximately 2-h work session on each day. During each 2-h session, the participant completed a series of four to seven assigned tasks (mean of five), each lasting from 5 to 20 min depending on the amount of time that the participant required to complete the task. Prior to the first session, the experimenter gave the participant a general description of the assessment (e.g., "I will present you with a series of work tasks to observe how you will carry them out. Please, feel free to complete your assignments as you like.") and instructed the participant to behave as if they were performing a real job.

Tasks were those commonly completed in office and retail positions, including folding or displaying clothing on shelves, tidying or storing items on shelves or in cabinets, filing documents, folding letters and putting them into envelopes, sorting items, stapling documents, shredding documents, tidying books or files, writing documents using Microsoft Word, entering data or creating graphs using Microsoft Excel, counting money in a cash box, creating educational materials (e.g., printing, cutting, laminating, and classifying flash cards), cleaning (e.g. vacuuming, drying tables), making photocopies, assembling boxes, and wrapping gifts.

When assigning each task, the experimenter who played the role of the "supervisor" provided the participant with instructions and materials consistent with the task. He waited 5 s before walking away or leaving the room to give the participant the opportunity to make a confirming statement and ask questions. The experimenter then either stated, "I'll be in my room if you need anything," before leaving the room and closing the door behind him or, "I will sit here at my table so I can keep working. I'm here, in case you need me,” before returning to his seat in the same room. If the participant made a confirming statement or asked a question, the experimenter responded accordingly. If the target responses were not emitted, the supervisor returned to the work room or approached the participant to provide feedback or to end the task if (1) the participant did not call or search for the supervisor within 1 min of stopping the assigned task; (2) worked unsuccessfully on the assigned task for 5 min; or (3) searched unsuccessfully for needed materials for 5 min.

The experimenter arranged one or more of the following evocative situations described by Lerman et al. (2017) and Grob et al. (2019) during each task such that the participant had four to seven opportunities to emit each target behavior during each 2-h session: (1) Clear instructions, during which the supervisor described and modeled how to complete the task; (2) vague instructions, during which the supervisor presented the participant with a new task that could be completed in multiple ways but did not describe or model how to complete it; (3) task not in the repertoire, during which the supervisor assigned the participant a task that required a skill not present in the participant's repertoire and did not describe or model how to complete it; (4) missing or broken materials, during which the supervisor did not provide the participant with all or enough materials to complete the task, or provided the necessary materials but some were broken; (5) task completed, during which the supervisor permitted the participant to complete all available tasks before returning to the work room; (6) clear corrective feedback, during which the supervisor explained why the work was not correct and demonstrated how to correct errors; and (7) vague corrective feedback, during which the supervisor told the participant that their work had to be corrected without specifying what was wrong or how to correct the mistake. The evocative situations were pseudo-randomized by adopting a random order of presentation while ensuring that the participant was not exposed to the same situation across two consecutive tasks to prevent predictability, possible sequence effects, or the generation of guiding rules.

### Intervention

Three target behaviors were selected for each participant based on the results of the assessment, shown in Table 3. Responses with asterisks were those targeted for each participant. The selected target behaviors occurred in less than 30% of the opportunities during the assessment. These targets were considered important for increasing the likelihood of success on the job (e.g., asking for help) and for improving social relationships with co-workers and supervisors (e.g., apologizing, asking what to do next).

**Table 3:** Percentage of Correct Responses during the Assessment for Each Participant

	Participant		
Behavior	Alberto	Maria	Giacomo
Confirm Understanding	14% (clear inst)* 90% (vague inst)	18% (clear inst)* 73% (vague inst)	9% (clear inst)* 19% (vague inst)
Ask for Help	90% (task) 90% (materials)	89% (task) 90% (materials)	27% (task) * 51% (materials)
Notify of Task Completion	96%	96%	94%
Ask for Task Assignment	3%*	0%*	51%
Respond to Corrective Feedback	0% (apologize)* 75% (ask for clear) 0% (confirm) 82% (correct error)	12% (apologize)* 100% (ask for clear) 12% (confirm) 93% (correct error)	0% (apologize)* 77% (ask for clear) 9% (confirm) 72% (correct error)

*Note*. Asterisks indicate the targets selected for intervention. Inst = instructions; task = ask for help with a task; materials = ask for help with materials; ask for clear = ask for clear feedback; confirm = confirm understanding of feedback

A mean of two sessions (range: 1–3) were conducted per week, each lasting no longer than 2 h, during which the participant was exposed to a series of assigned tasks, each lasting about 10 min to 15 min. The experimenter presented five to seven opportunities for the participant to emit the targeted behaviors during each session by arranging the relevant evocative situations as described previously.

#### Training

The goal of the initial training was to evaluate the effects of BST, prompts, praise, feedback, and a token economy on skill acquisition. A multiple baseline design across behaviors was used to evaluate the effects of the intervention on skill acquisition. During training, the experimenter conducted a brief BST session prior to each work session using procedures described by Grob et al. (2019). First, the experimenter provided a rationale for exhibiting the target behavior and described how to do so. For example, when teaching the participant to ask for their next task assignment, the experimenter described how and when the participant should ask for their next assignment and explained why it was important to do so. Next, the experimenter modeled some examples of correct and incorrect responses. The experimenter then had the participant practice the targeted skill within the context of the relevant situation while the experimenter provided immediate praise and descriptive feedback contingent on correct responses. If the participant performed the behavior incorrectly, the experimenter provided immediate corrective feedback by describing the error and what the participant should have done instead and then arranged additional opportunities for the participant to practice the behavior until they emitted the response correctly. The experimenter terminated the BST when the participant emitted the correct response once. None of the participants had to practice the skill more than twice before proceeding to the work session, which commenced after a 5-min break.

During each work session, the experimenter assigned tasks as described previously. The duration of each work session was comparable to the assessment phase. However, the experimenter told the participant that they would receive a token each time they correctly exhibited the behavior practiced during BST. All participants were already familiar with using a token economy because it was a common strategy included in their behavior plans for skill acquisition. Tokens earned during the job-related social skill training remained separate from those earned in other acquisition programs. Contingent on each correct response that was not preceded by a prompt, the experimenter immediately delivered one token, praise, and descriptive feedback (e.g., “Excellent, you earned a point because you apologized without getting angry,” or “That’s cool, you repeated a part of the instruction that I provided.”). If the participant did not exhibit the correct response, the experimenter immediately modeled the correct response and instructed the participant to practice until they engaged in the correct response. The experimenter delivered praise for correct responses that followed a model. The participant could exchange five tokens for an item or activity of their choice (e.g., walk or listen to music with the supervisor, a snack at the cafeteria, video call with friends) at the end of the session. This training package continued until the participant engaged in the correct response during at least 80% of the opportunities for three consecutive sessions.

#### Fading

The purpose of this phase was to begin fading the intervention to help promote maintenance and generalization of the targeted skills. To do so, the experimenter removed some of the training components. Sessions were continued as described in training except that the experimenter no longer preceded each work session with BST or delivered tokens for correct responses. The experimenter provided only immediate praise and descriptive feedback contingent on correct responses. Contingent on incorrect responses, the experimenter described what the participant did incorrectly, modeled the correct response, and gave the participant an opportunity to practice the targeted skill. This phase continued until the participant emitted each skill during at least 80% of opportunities for three consecutive sessions. Fading was omitted for the third skill targeted for each participant to test whether a quicker transition to the extension phase was possible. If there was a decrease in performance, the experimenter would have conducted additional training for the third skill but this was not necessary.

### Extension

The purpose of this phase was to evaluate the generality of the training by extending it to contexts beyond a controlled clinic setting. The experimenter evaluated the participant’s performance with an abbreviated version of the original intervention in a different setting and with a different supervisor and tasks described previously. Alberto’s extension sessions occurred immediately after his intervention phase. However, closures and restrictions associated with the COVID-19 pandemic resulted in substantial delays between the end of the fading phase and the initiation of extension sessions for Maria and Giacomo (5 months for Maria and 13 months for Giacomo). The experimenter implemented a single “booster” BST session with each participant for each target behavior in the new setting prior to the first work session using the same procedures described previously. The purpose of the booster BST session was to decrease the likelihood of errors and facilitate the transition to a new working environment, which was considered particularly important due to the pandemic-related disruptions in the opportunity to emit the responses. A BST booster also was conducted if correct responding decreased below 50% of opportunities for a targeted skill. All other procedures were identical to those in baseline.

The extension sessions were conducted in real work settings, as described previously, which included a toy and bookstore for children (for Maria and Giacomo) and a professional school for catering (for Alberto). However, due to restrictions associated with the COVID-19 pandemic, Maria and Giacomo also received extension sessions in a psychotherapy office at the center (located in a different building than the training setting) at various times during this phase. For Maria, the transition to the psychotherapy office occurred 13 months after she had received four sessions in the toy and bookstore. She was then able to return to the toy and bookstore after four sessions in the psychotherapy office. Giacomo’s initial extension sessions occurred in the psychotherapy office. His transition to the toy and book store occurred 4 months after he received three sessions in the psychotherapy office.

An experimenter who did not participate in any prior sessions with the participants served as the supervisor in the psychotherapy office setting. The supervisors in the toy and bookstore and catering settings were individuals who worked in those settings. Prior to the sessions, the experimenter trained the supervisors how to implement the relevant evocative situations. The experimenter also was present during the sessions to collect data and ensure that the supervisor implemented the procedures with fidelity, but the experimenter never interacted with the participant. The experimenter always remained at least 4 m from the participant so that the participant could not hear any instructions or feedback provided to the supervisor.

### Social Validity

After the study was completed, the experimenter assessed the social validity of the targets and the intervention by sending a survey to the caregivers of the participants, caregivers of individuals who were of similar ages and adaptive skill levels as the participants, and professionals employed at the center where the study took place. The survey items are displayed in Table 4. The experimenter sent a link to an electronic version of the survey (in a Google Sheets spreadsheet) via email to a total of seven caregivers and six professionals. Before starting the survey, the respondents were instructed to read the following:

We are interested in your opinion about various social skills that employees might demonstrate on the job and about various interventions for teaching social skills. This survey will take approximately 5 minutes to complete. All responses will remain anonymous and confidential. We will never link responses to people that completed the survey.

**Table 4:** Median Ratings (with Ranges) of Caregivers and Professionals on the Social Validity Survey

Below, we list different social skills and ask you to rate the importance of this skill for the employees using a scale from 1 (not at all important) to 5 (extremely important). That is, how important do you feel this skill is for employees to be successful on the job?		
Item	Caregivers	Professionals
The employee confirms that he understands that a task has been assigned to him (e.g. "I get it!"; "Okay, I'll do it!).	4.0 (3–5)	4.0 (3–5)
The employee requests a demonstration when unable to complete a task.	4.0 (3–5)	4.0 (3–5)
The employee asks the supervisor for the next task when he has finished the previous one.	4.0 (2–4)	4.0 (3–5)
The employee accepts the employer's corrections with respect to their mistakes (e.g., "Thanks for pointing it out"; "Sorry for the mistake").	5.0 (2–5)	4.0 (4–5)
Below, we list different interventions for teaching social skills and ask you to rate the acceptability of intervention using a scale from 1 (not at all acceptable) to 5 (extremely acceptable)		
Item	Caregivers	Professionals
Describing the skill and then practicing it in role play.	4.0 (3–4)	4.0 (2–5)
Praising correct responses.	4.0 (n/a)	5.0 (4–5)
Delivering tokens for correct responses.	4.0 (2–4)	4.0 (3–5)
Delivering feedback for errors.	4.0 (3–4)	5.0 (4–5)

Respondents provided their answers directly on the form by selecting the cell corresponding to the preferred answer. All seven caregivers and five of the professionals completed the survey.

The experimenter also asked the three participants to complete a different survey to evaluate the acceptability of the procedures and outcomes. The survey items are displayed in Table 5. The experimenter administered the survey individually to each participant by displaying it on a computer screen, reading each item aloud to the participant, answering any of their questions by providing examples, and asking the participant to select their responses using the trackpad of the laptop.

**Table 5:** Participant Ratings on Social Validity Survey

Please rate the following aspects of the training you received when you learned how to interact better with supervisors in the workplace. Indicate how much you agree or disagree with each of the statements			
Item	Alberto	Maria	Giacomo
I liked learning how to interact with my supervisor at work.	4	3	5
The training helped me learn how to interact with my supervisor, such as apologizing when I made mistakes and letting my supervisor know that I understood the instructions.	5	4	5
I enjoyed earning tokens when I did well.	5	5	5
I enjoyed receiving feedback about my performance.	5	1	5
I enjoyed practicing the skills with my experimenter.	4	5	5
I would recommend this program to others.	4	4	5

*Note*. 1 = disagree, 2 = somewhat disagree, 3 = neither agree nor disagree, 4 = somewhat agree, 5 = agree

## Results

**Fig. 1:**
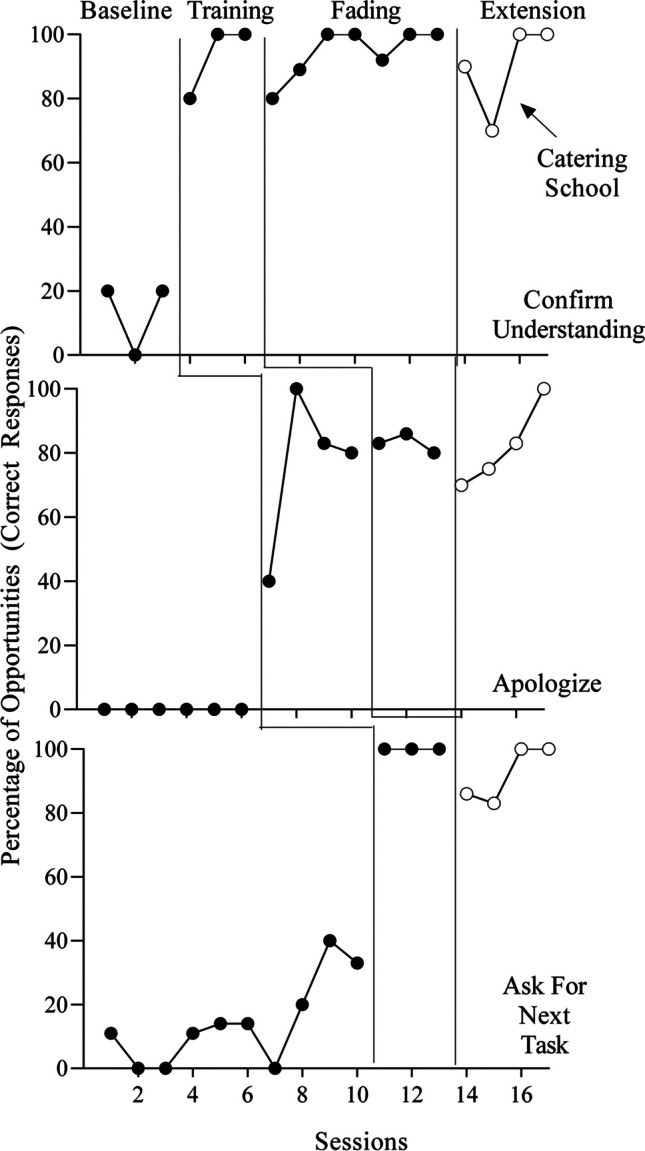
Percentage of Correct Responses for Alberto

**Fig. 2:**
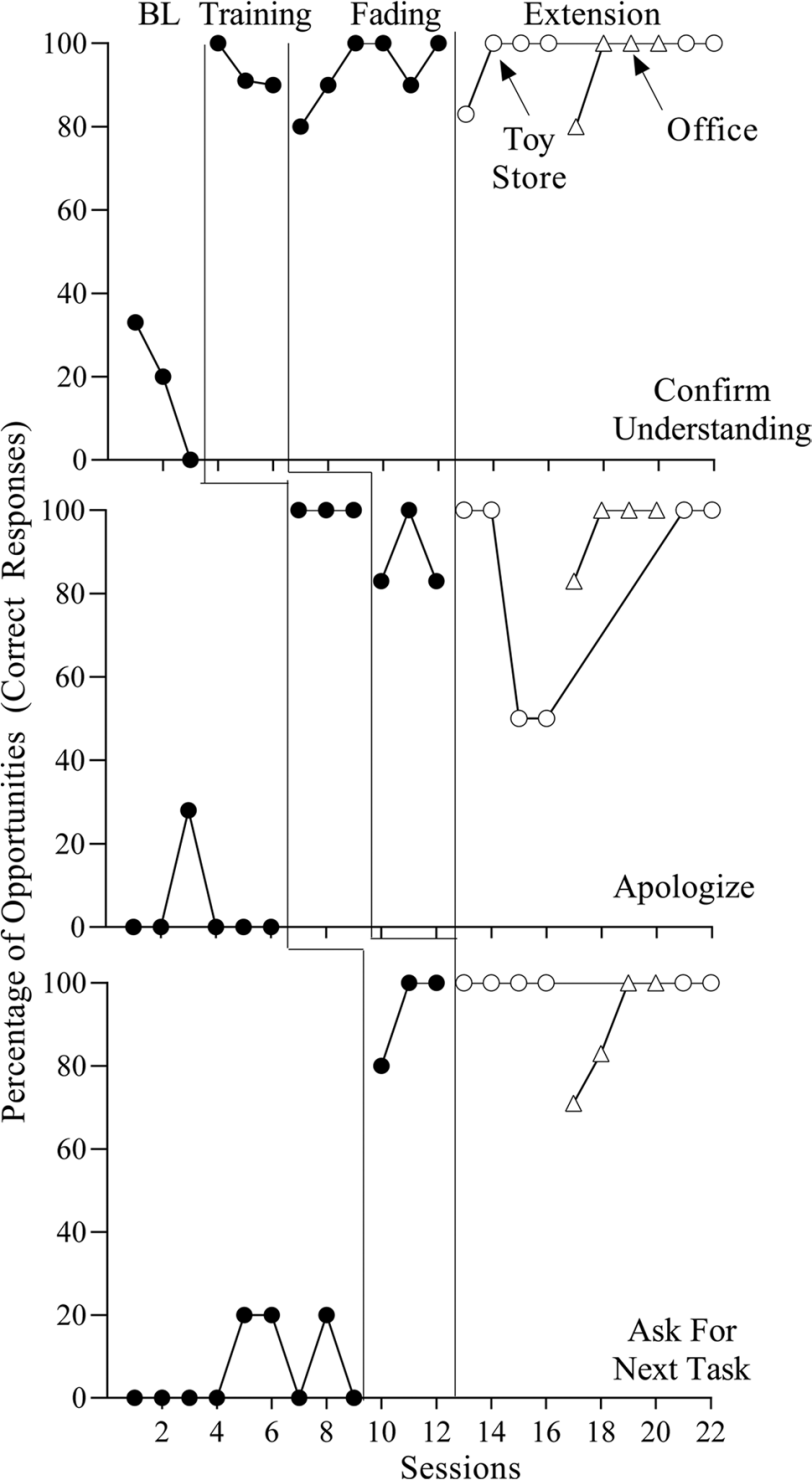
Percentage of Correct Responses for Maria. *Note*. BL = Baseline

**Fig. 3:**
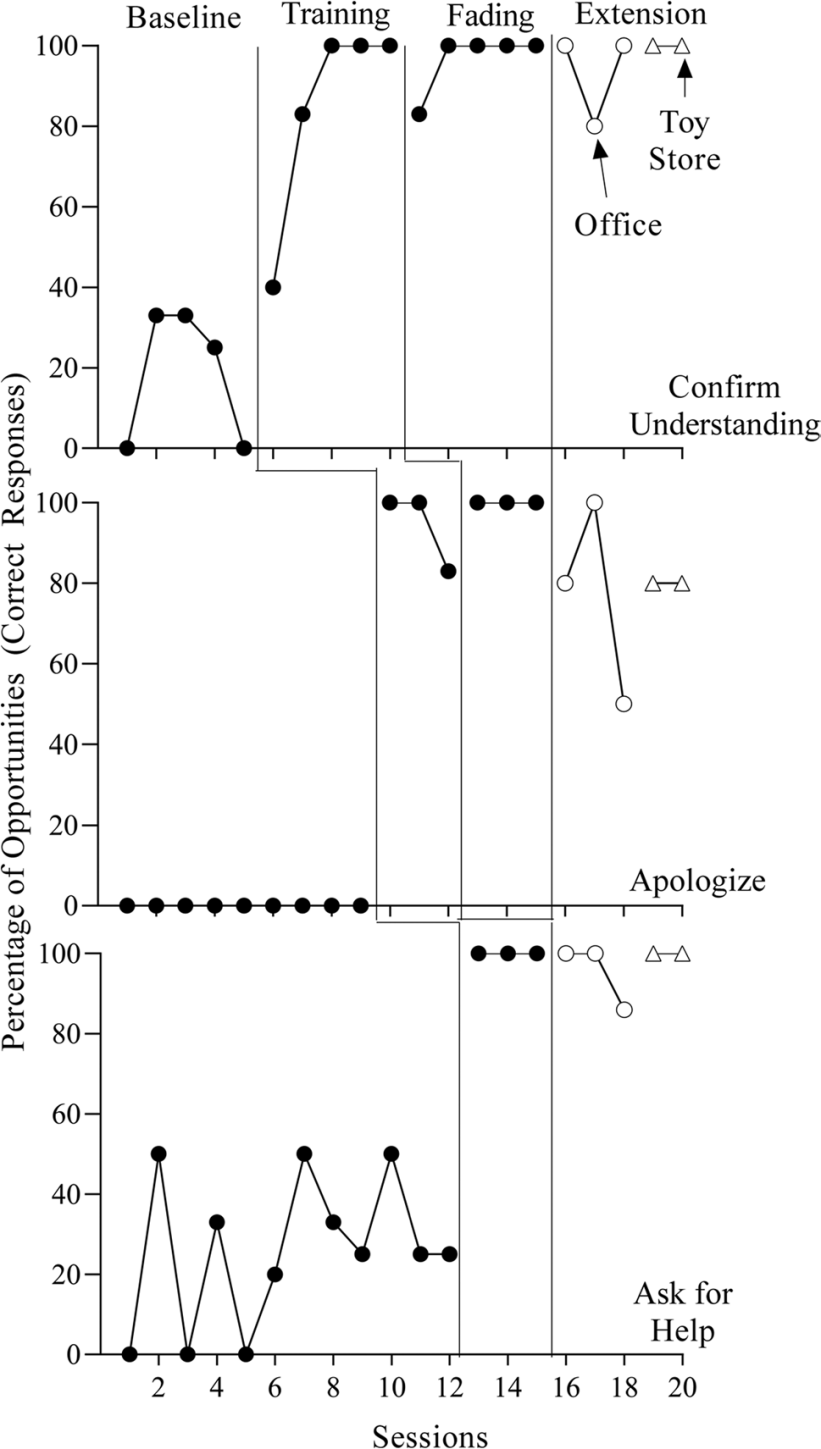
Percentage of Correct Responses for Giacomo

Results for the three participants are displayed in Figs. 1, 2, and 3. Alberto (Fig. 1) engaged in either zero or low levels of the targeted responses in baseline. Initial training produced fairly immediate increases in correct responding for all targets (*M* = 93% [confirm understanding], 76% [apologize], 100% [ask for next task]). Performance remained high when the experimenter removed some of the training components (designated as “fading” on the graphs) for the first two targets (*M* = 94% [confirm understanding], 83% [apologize]). Following the BST booster at the catering school, Alberto’s responding remained high for all three targets (*M* = 90% [confirm understanding], 82% [apologize], 92% [ask for next task]).

Maria (Fig. 2) engaged in either zero or low levels of the targeted responses in baseline. Like Alberto, initial training produced fairly immediate increases in Maria’s correct responding for all targets (*M* = 94% [confirm understanding], 100% [apologize], 93% [ask for next task]). Performance remained high when the experimenter removed some of the training components (designated as “fading” on the graphs) for the first two targets (*M* = 93% [confirm understanding], 89% [apologize]). Following the BST booster in the toy store, Maria’s performance remained high with the exception of one target (apologizing), which decreased to 50% of opportunities in sessions 15 and 16 (*M* = 96% [confirm understanding], 75% [apologize], 100% [ask for next task]). Thus, Maria received a BST booster for this target immediately prior to session 17 (in the second setting, the psychotherapy office) due to the decrease in apologizing and the significant passage of time between sessions 16 and 17 (13 months). Maria’s apologizing immediately increased in the psychotherapy office (*M* = 95% [confirm understanding], 96% [apologize], 88% [ask for next task]) and remained high for all three targets when she transitioned back to the toy store (*M* = 100% for all targets].

Like the other participants, Giacomo’s levels of the targeted responses (Fig. 3) were somewhat low in baseline, quickly increased during the initial training (*M* = 85% [confirm understanding], 94% [apologize], 100% [ask for help]), and remained high when the experimenter removed some of the training components for two targets (*M* = 97% [confirm understanding], 100% [apologize). Following the BST booster in the psychotherapy office, Giacomo’s performance remained high with the exception of apologizing (*M* = 93% [confirm understanding], 77% [apologize], 95% [ask for help]). Similar to Maria, this target decreased to 50% of opportunities in session 18. Thus, Giacomo received a BST booster for this target immediately prior to session 19 (in the second setting, the toy store) due to the decrease in apologizing and the significant passage of time between the two sessions (4 months). Giacomo’s performance was high during the two sessions in the toy store for all three targets (*M* = 100% [confirm understanding], 80% [apologize], 100% [ask for help]).

Median responses of the caregivers and professionals on the social validity survey are shown in Table 3. The majority of caregivers rated all of the targets as fairly important (4.0) to extremely important (5.0), with the lowest ratings assigned to apologizing for or acknowledging mistakes and confirming understanding of instructions. Caregiver ratings of the intervention components indicated that they found them to be somewhat acceptable (4.0) or very acceptable (5.0), with the lowest ratings assigned to delivery of tokens and the highest ratings assigned to delivery of praise. The professionals tended to provide slightly higher ratings than did caregivers for the targets and the intervention components.

Responses of the three participants on their social validity survey are displayed in Table 4. Alberto and Giacomo either somewhat agreed or agreed with all of the statements on the survey, indicating that they liked the training and thought it was helpful. Maria somewhat agreed or agreed with all but two of the statements. She neither agreed nor disagreed that she liked learning how to interact with her supervisor at work, and she disagreed that she enjoyed receiving feedback about her performance.

## Discussion

Three adults with ASD residing in Italy showed rapid acquisition of job-related social skills in both clinic-based and real work settings after receiving a package of commonly used behavior-analytic interventions, including BST and token reinforcement. Despite lengthy disruptions to their work schedules as a result of the COVID-19 pandemic, the participants continued to perform the skills at high levels in real work contexts with brief BST boosters. Overall feedback provided by the participants, caregivers, and professionals suggested that they found the targets and intervention components to be acceptable. Together, these findings replicate and extend prior research conducted in the United States (e.g., Grob et al., 2019; Lerman et al., 2017), providing initial evidence of the cultural relevance of both the targeted skills and the interventions for those residing in Italy. The inclusion of these participants was particularly important because traditional education both for typically developing and for students with NDD in Italy tends to focus on academic skills rather than the skills needed to successfully obtain and maintain employment (Pastore, 2018).

Although a number of studies have evaluated protocols for teaching job skills, the literature contains relatively little research on best practices for teaching social skills needed in the workplace (see Campanaro et al., 2021, for a review). Given that job-related social skills can be particularly challenging for employees with NDD, further research in this area might lead to improved employment outcomes for this population. This study, together with that of Grob et al. (2019) and by Lerman et al. (2017), represents a contribution in this direction.

The low employment rate of individuals with NDD is a global problem (Cameron et al., 2022; Howlin & Moss, 2012; Maslahat et al., 2022; Roux et al., 2013; Wei et al., 2015; Zhou et al., 2022). Thus, further research is needed in a variety of countries and cultures to identify effective and acceptable solutions to this problem and to disseminate knowledge that would improve the lives of individuals with NDD across the globe. The specific behaviors targeted in this study were selected due to their relevance to successful completion of responsibilities (e.g., asking for help) and to their potential role in building good social relationships (e.g., apologizing). However, a myriad of skills, such as interviewing for jobs, solving workplace problems, and managing time, are needed to obtain and maintain employment. Moreover, future researchers could evaluate whether specific vocational skills protocols promote a higher rate of employment by carrying out feasibility trials across a larger number of workers with NDD and work settings. In addition to interventions for these skills, researchers should focus on strategies for increasing employers’ knowledge about NDD, their willingness to hire workers with NDD, and their preparedness to arrange accommodations that would help ensure their workers’ success on the job.

Further research also is needed to determine the most effective approaches for promoting maintenance and generalization of job skills, with a particular focus on the transition from school or clinics to employment settings. In the current study, one skill (apologizing) did not maintain in the real work setting for two participants, a problem that a single BST booster appeared to remedy. These findings suggest that fading intervention components and providing brief BST boosters, as needed, may be a viable approach for ensuring that skills taught in a school or clinic setting transfer to work settings and maintain over time. However, this conclusion must remain tentative due to several limitations. First, the intervention consisted of multiple components, including BST, token reinforcement, feedback, and fading in the training setting, along with brief BST boosters in the work settings. Thus, a component analysis would be needed to identify the necessary and sufficient elements of this package.

A second limitation is that the experimenter did not remove BST and token reinforcement for one of the three targeted skills prior to the extension phase, raising questions about the necessity of fading. Third, the experimenter’s presence in the extension setting may have at least partially controlled the participants’ performance. Fourth, COVID-related interruptions in work and training schedules prevented the experimenter from evaluating long-term maintenance in a systematic, controlled manner. Finally, none of the participants’ performance was evaluated in the real work settings prior to intervention. Baseline levels of responding in those settings were needed to evaluate the effects of the training on performance in those settings.

Further replications and extensions of this work should explore these additional research questions, as the results may have a meaningful impact on employment outcomes for individuals with NDD throughout the world. The benefits of regular employment are substantial for these individuals and their communities. Filling serious gaps in our knowledge about how best to promote successful outcomes in this area is needed before we can establish an effective technology of behavior change.

## Data Availability

The data that support the findings of this study are openly available in google drive at https://drive.google.com/drive/folders/1MyRVQha10l4J-QNLO06zOARf8i82ui87?usp=sharing. The authors confirm that the data supporting the findings of this study are available within the article.

## References

[CR1] American Psychiatric Association. (2000). *Diagnostic and statistical manual of mental disorders* (4th ed., Text Rev.).

[CR2] Anderson, A., Moore, D. W., Rausa, V. C., Finkelstein, S., Pearl, S., & Stevenson, M. (2017). A systematic review of interventions for adults with autism spectrum disorder to promote employment. *Review Journal of Autism & Developmental Disorders,**4*(1), 26–38. 10.1007/s40489-016-0094-9

[CR3] Baranger, A. (2019, November 5). State of play of employment of people on the autism spectrum in Europe: Barriers, good practices and trends [paper presentation]. Committee on Employment and Social Affairs of the European Parliament. https://www.autismeurope.org/wp-content/uploads/2019/11/presentation_employment_autism_final2.pptx.pdf

[CR4] Barbaro, D., & Shankardass, K. (2022). Work-related social skills interventions for individuals with autism spectrum disorder throughout the life course. *Review Journal of Autism & Developmental Disorders*. 10.1007/s40489-022-00317-7

[CR5] Bennett, K. D., & Dukes, C. (2013). A systematic review of teaching daily living Skills to adolescents and adults with autism spectrum disorder. *Review Journal of Autism & Developmental Disorders,**1*(1), 2–10. 10.1007/s40489-013-0004-3

[CR6] Brand, J. E. (2015). The far-reaching impact of job loss and unemployment. *Annual Review of Sociology,**41*(1), 359–375. 10.1146/annurev-soc-071913-043237 26336327 10.1146/annurev-soc-071913-043237 PMC4553243

[CR7] Cameron, L. A., Tonge, B. J., Howlin, P., Einfeld, S. L., Stancliffe, R. J., & Gray, K. M. (2022). Social and community inclusion outcomes for adults with autism with and without intellectual disability in Australia. *Journal of Intellectual Disability Research,**66*(7), 655–666. 10.1111/jir.12953 35677963 10.1111/jir.12953 PMC9328353

[CR8] Campanaro, A. M., Vladescu, J. C., Manente, C. J., Deshais, M. A., & DeBar, R. M. (2021). A review of the literature on vocational training interventions with individuals with autism spectrum disorder. *Behavioral Interventions,**36*(3), 675–696. 10.1002/bin.1795

[CR9] Fleming, A. R., Fairweather, J. S., & Leahy, M. J. (2013). Quality of life as a potential rehabilitation service outcome: The relationship between employment, quality of life, and other life areas. *Rehabilitation Counseling Bulleting,**57*(1), 9–22. 10.1037/0021-9010.90.1.53

[CR10] García-Villamisar, D., Wehman, P., & Navarro, M. (2002) Changes in the quality of autistic people’s life that work in supported and sheltered employment. A 5-year follow-up study. *Journal of Vocational Rehabilitation*, *17*(4), 309–312. https://content.iospress.com/articles/journal-of-vocational-rehabilitation/jvr00170?resultNumber=0&totalResults=507&start=0&q=Changes+in+the+quality+of+autistic+people%E2%80%99s+life+that+work+in+supported+and+sheltered+employment.+A+5-year+follow-up+study&resultsPageSize=10&rows=10

[CR11] Gorenstein, M., Giserman-Kiss, I., Feldman, E., Isenstein, E. L., Donnelly, L., Wang, A. T., & Foss-Feig, J. H. (2020). Brief report: A Job-Based Social Skills Program (JOBSS) for adults with autism spectrum disorder: A pilot randomized controlled trial. *Journal of Autism and Developmental Disorders,**50*(12), 4527–4534. 10.1007/s10803-020-04482-8 32297122 10.1007/s10803-020-04482-8

[CR12] Greenspan, S., & Shoultz, B. (1981). Why mentally retarded adults lose their jobs: Social competence as a factor in work adjustment. *Applied Research in Mental Retardation*, *2*(1), 23–38. 10.1016/0270-3092(81)90004-7 10.1016/0270-3092(81)90004-7 7305328

[CR13] Grob, C. M., Lerman, D. C., Langlinais, C. A., & Villante, N. K. (2019). Assessing and teaching job-related social skills to adults with autism spectrum disorder. *Journal of Applied Behavior Analysis,**52*(1), 150–172. 10.1002/jaba.503 30221363 10.1002/jaba.503

[CR14] Hendricks, D. (2010). Employment and adults with autism spectrum disorders: Challenges and strategies for success. *Journal of Vocational Rehabilitation*, *32*(2), 125–134. https://doi:10.3233/JVR-2010-0502

[CR15] Howlin, P., & Moss, P. (2012). Adults with autism spectrum disorders. *Canadian Journal of Psychiatry,**57*(5), 275–283. 10.1177/070674371205700502 22546059 10.1177/070674371205700502

[CR16] Kellems, R. O., & Morningstar, M. E. (2012). Using video modeling delivered through iPods to teach vocational tasks to young adults with autism spectrum disorders. *Career Development & Transition for Exceptional Individuals,**35*(3), 155–167. 10.1177/0885728812443082

[CR17] Lerman, D. C., White, B., Grob, C., & Laudont, C. (2017). A clinic-based assessment for evaluating job- related social skills in adolescents and adults with autism. *Behavior Analysis in Practice,**10*(4), 323–336. 10.1007/s40617-017-0177-9 29214128 10.1007/s40617-017-0177-9 PMC5711736

[CR18] McKee-Ryan, F., Song, Z., Wanberg, C. R., & Kinicki, A. J. (2005). Psychological and physical well-being during unemployment: A meta-analytic study. *Journal of Applied Psychology,**90*(1), 53–76. 10.1037/0021-9010.90.1.53 15641890 10.1037/0021-9010.90.1.53

[CR19] Maslahati, T., Bachmann, C. J., Höfer, J., Küpper, C., Stroth, S., Wolff, N., Poustka, L., Roessner, V., Kamp-Becker, I., Hoffmann, F., & Roepke, S. (2021). How do adults with autism spectrum disorder participate in the labor market? A German multi-center survey. *Journal of Autism & Developmental Disorders,**52*(3), 1066–1076. 10.1007/s10803-021-05008-6 33864556 10.1007/s10803-021-05008-6 PMC8854283

[CR20] Montague, M., Lund, K., & Warner, C. (2017). *Job-related social skills: A curriculum* (3^rd^ ed). Exceptional Innovations.

[CR21] Montgomery, J., Storey, K., Post, M., & Lemley, J. (2011). The use of auditory prompting systems for increasing independent performance of students with autism in employment training. *International Journal of Rehabilitation Research,**34*(4), 330–335. 10.1097/MRR.0b013e32834a8fa8 21885987 10.1097/MRR.0b013e32834a8fa8

[CR22] Morgan, L., Leatzow, A., Clark, S., & Siller, M. (2014). Interview skills for adults with autism spectrum disorder: A pilot randomized controlled trial. *Journal of Autism & Developmental Disorders,**44*(9), 2290–2300. 10.1007/s10803-014-2100-3 24682707 10.1007/s10803-014-2100-3

[CR23] Mueller, H. H. (1988). Employer's reasons for terminating the employment of workers in entry-level jobs: Implications for workers with mental disabilities. *Canadian Journal of Rehabilitation, 1*(4), 233–240. https://psycnet.apa.org/record/1989-37861-001

[CR24] Office for National Statistics. (2022). *Disability and employment, UK, 2021.* https://www.ons.gov.uk/peoplepopulationandcommunity/healthandsocialcare/disability/datasets/disabilityandemployment

[CR25] Partington, J. W., & Mueller, M. M. (2015). The assessment of functional living skills vocational skills assessment protocol: An assessment, skills tracking system, and curriculum guide for skills that are essential for independence. Behavior Analysts/Stimulus Publications.

[CR26] Pastore, F. (2018). Why so slow? The school-to-work transition in Italy. *Studies in Higher Education,**44*(8), 1358–1371. 10.1080/03075079.2018.1437722

[CR27] Radley, K. C., Dart, E. H., Brennan, K. J., Helbig, K. A., Lehman, E. L., Silberman, M., & Mendanhall, K. (2020). Social Skills Teaching for individuals with autism spectrum disorder: A systematic review. *Advances in Neurodevelopmental Disorders,**4*(3), 215–226. 10.1007/s41252-020-00170-x

[CR28] Roid, G., & Miller, L. (1997). *Leiter International Performance Scale-Revised*. Stoelting.

[CR29] Roux, A. M., Shattuck, P. T., Cooper, B. P., Anderson, K. A., Wagner, M., & Narendorf, S. C. (2013). Postsecondary employment experiences among young adults with an autism spectrum disorder. *Journal of the American Academy of Child & Adolescent Psychiatry,**52*(9), 931–939. 10.1016/j.jaac.2013.05.019 23972695 10.1016/j.jaac.2013.05.019 PMC3753691

[CR30] Taylor, J. L., Smith, L. E., & Mailick, M. R. (2014). Engagement in vocational activities promotes behavioral development for adults with autism spectrum disorders. *Journal of Autism & Developmental Disorders,**44*(6), 1447–1460. 10.1007/s10803-013-2010-9 24287880 10.1007/s10803-013-2010-9 PMC4024367

[CR31] Watanabe, M., & Sturmey, P. (2003). The effect of choice-making opportunities during activity schedules on task engagement of adults with autism. *Journal of Autism & Developmental Disorders,**33*(5), 535–538. 10.1023/A:1025835729718 14594333 10.1023/a:1025835729718

[CR32] Wechsler, D. (1981). *Wechsler Adult Intelligence Scale* (rev.). The Psychological Coro.

[CR33] Wei, X., Wagner, M., Hudson, L., Yu, J. W., & Shattuck, P. (2015). *Transition to Adulthood. Emerging Adulthood,**3*(1), 37–45. 10.1177/2167696814534417

[CR34] Zhou, L., Wang, J., & Huang, J. (2021). Brief report: Health expenditures for children with autism and family financial well-being in China. *Journal of Autism & Developmental Disorders,**52*(8), 3712–3717. 10.1007/s10803-021-05214-2 34318432 10.1007/s10803-021-05214-2

